# Supplementary material for: Evidence supported by Mendelian randomization: impact on inflammatory factors in knee osteoarthritis
Source: Front Med (Lausanne). 2024 May 28;11:1382836. doi: 10.3389/fmed.2024.1382836 (PMC11165061; doi:10.3389/fmed.2024.1382836)
Supplement: Supplementary file 1 [file Data_Sheet_1.ZIP › data/GCST90274806_5e6/806.forest.pdf]

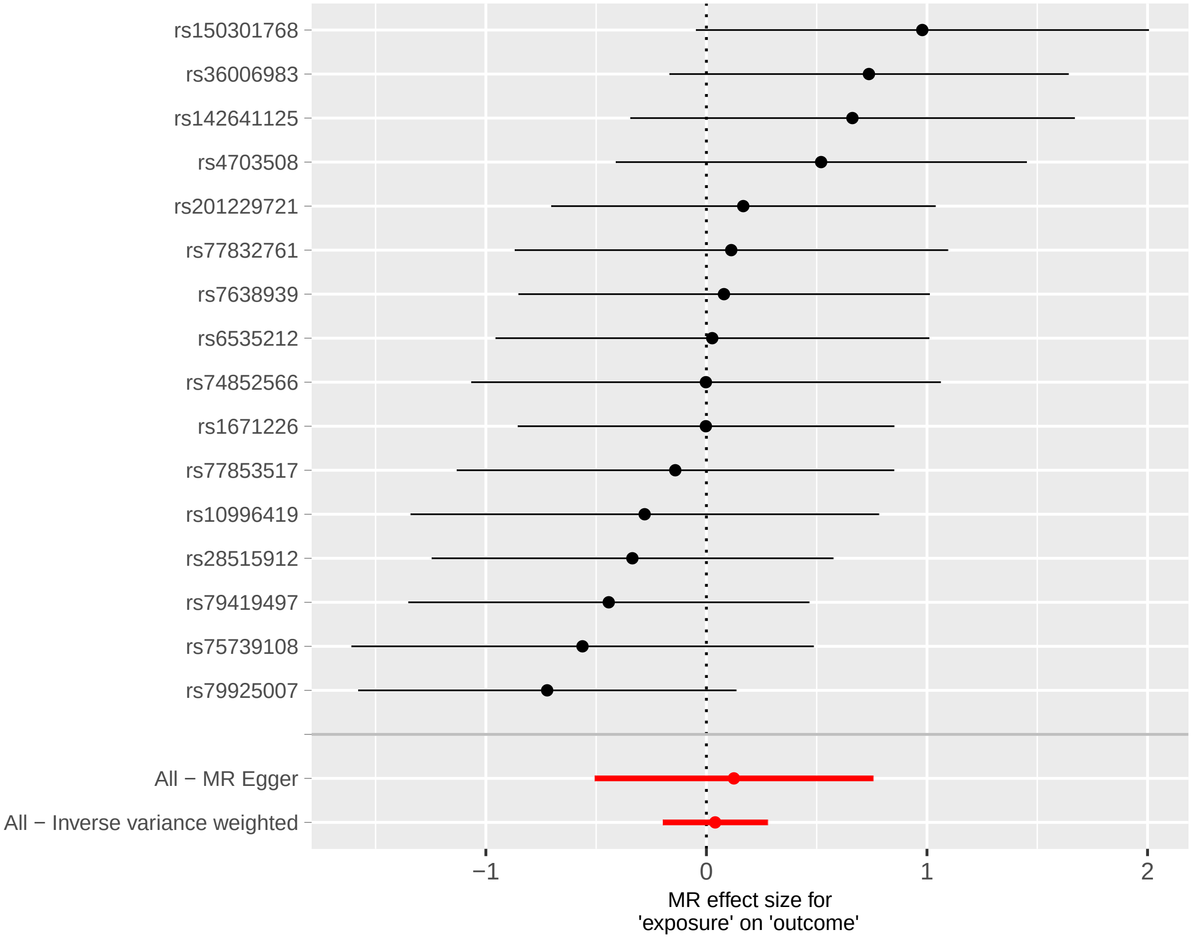
rs150301768
rs36006983
rs142641125
rs4703508
rs201229721
rs77832761
rs7638939
rs6535212
rs74852566
rs1671226
rs77853517
rs10996419
rs28515912
rs79419497
rs75739108
rs79925007
All − MR Egger
All − Inverse variance weighted
−1
0
1
2
MR effect size for
'exposure' on 'outcome'
